# Perennial Ryegrass Alkaloids Increase Respiration Rate and Decrease Plasma Prolactin in Merino Sheep under Both Thermoneutral and Mild Heat Conditions

**DOI:** 10.3390/toxins11080479

**Published:** 2019-08-19

**Authors:** Michelle L. E. Henry, Stuart Kemp, Iain J. Clarke, Frank R. Dunshea, Brian J. Leury

**Affiliations:** 1 Faculty of Veterinary and Agricultural Sciences, University of Melbourne, Melbourne, VIC 3010, Australia; 2 Gundagai Meat Processors, 2916 Gocup Rd, South Gundagai, NSW 2722, Australia; 3 PastureWise Pty. Ltd., 1485 Bamganie Rd, Cargerie, VIC 3334, Australia

**Keywords:** sheep, heat stress, ergovaline, lolitrem B, production, physiology, staggers

## Abstract

A study was undertaken to determine the effects of feeding two levels of perennial ryegrass alkaloids (nil vs. moderate) under two climatic conditions. Alkaloids were fed via endophyte-infected perennial ryegrass seed and hay. Twenty-four Merino ewe weaners (six months, initial BW 32 ± 1.7 kg) were used in a study that lasted for 21 days after 14 days of adaptation. Sheep were fed either a control or alkaloid (Alk, 110 μg/kg LW ergovaline and 75 μg/kg LW lolitrem B) supplemented diet. Sheep were exposed to either constant thermoneutral (TN, 21–22 °C, 49% RH) or mildly heated (HS, 33 °C 1000–1500 h, 28% relative humidity) conditions. Dietary Alk and HS reduced dry matter intake (DMI) (*p* < 0.001, *p* = 0.02, respectively) with the combination of both reducing DMI by 42%. Reductions in DMI resulted in a lower daily gain in the Alk treatment (*p* < 0.001). Feed digestibility was reduced in the combined treatment (*p* = 0.03). Rectal temperature, respiration rate, and skin temperature increased in the Alk treatment. Plasma prolactin concentrations were decreased by Alk and increased by mild HS. The data indicate that production is compromised in the presence of Alk and mild HS, with this effect being exacerbated by a combination of both.

## 1. Introduction

Perennial ryegrass (*Lolium perenne*) is a persistent and productive pasture grass found in the cool temperate, winter-spring rainfall region of Australia [1]. An internal fungus, referred to as an endophyte (*Neotyphodium lolli*) lives in a symbiotic relationship with the plant, producing alkaloids, including ergovaline and lolitrem B, during times of the year when plants are under stress. When ingested in high levels, these alkaloids can result in grazing animals developing perennial ryegrass toxicity. Lolitrem B is an indole-diterpenoid, is neurotoxic and has been shown to adversely impact many biological systems, including gut motility and gastrointestinal function [2]. It is the main alkaloid responsible for the development of tremors and staggering in sheep [3], and this has been well documented in New Zealand [3,4] and Australia [1]. Ergovaline ingestion is associated with elevated rectal temperature [4,5,6,7,8], reduced prolactin secretion in sheep [5] and may reduce animal production [1,4]. 

The temperate regions of Australia where perennial ryegrass (PRG) is the predominant pasture grass species experience a variable climate that is often characterized by large variations in ambient temperature during summer and autumn. Consumption of PRG can cause hyperthermia in a dose-dependent manner under thermoneutral [5] and high heat stress (HS) conditions [6], but very little is known about the potential interactions between PRG alkaloid ingestion and mild HS conditions, as commonly observed in summer and autumn when PRG alkaloids can be high. Therefore, the aim of this study was to investigate the interaction between PRG alkaloid ingestion and mild HS in Merino ewe weaners by measuring the production, physiological, metabolic, and hormonal responses.

## 2. Results

### 2.1. Environmental Conditions and Production Responses

Ambient temperature increased by an average of 12 °C during the heated period in the Heat groups and remained at constant thermoneutral (TN) conditions in the TN groups (average 21–22 °C). Under the heat conditions the average value for peak temperature-humidity index (THI) was 28.7 between 1100 and 1600 h indicating mild heat stress conditions [9] and was 20.0 between 1800 and 0900 h. During TN conditions, the average THI was 19.8. There were no visible signs of PRG toxicity or staggers at any stage of the study.

**Figure 1:**
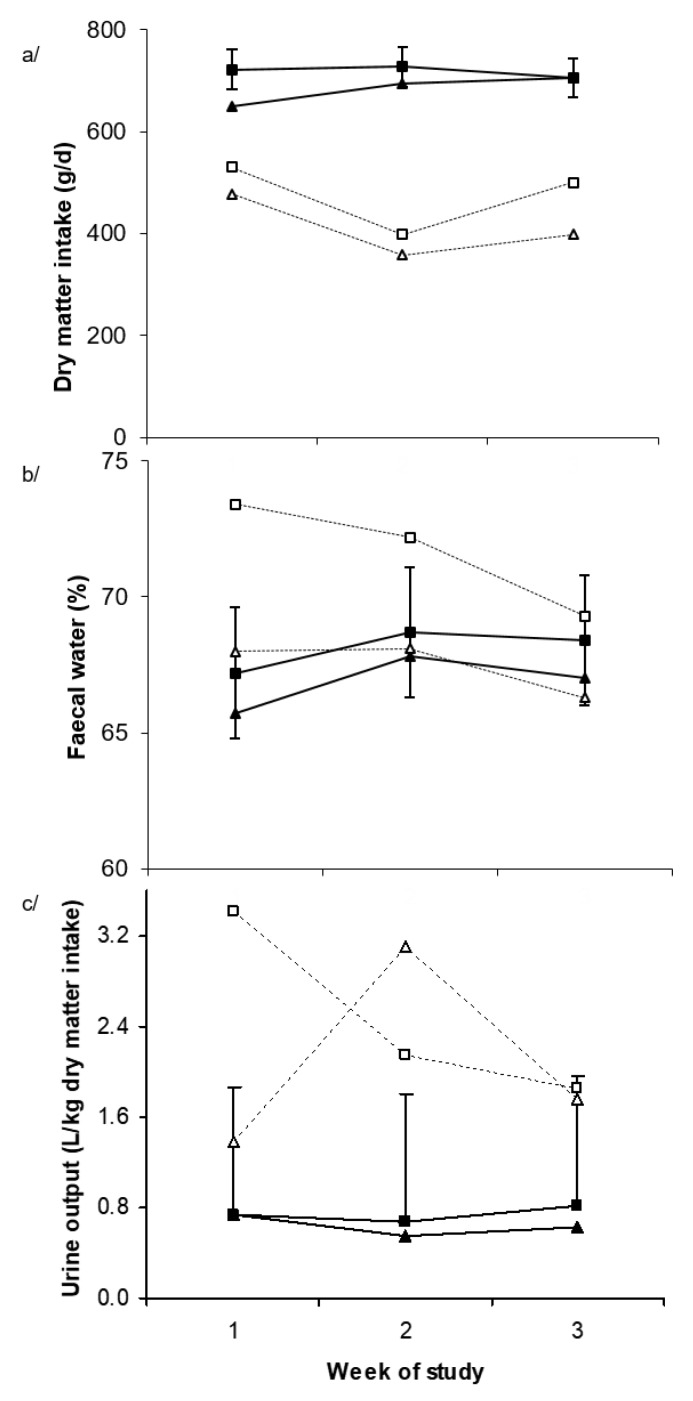
(**a**) Dry matter intake, (**b**) fecal water, and (**c**) urine output for each week of study in Merino sheep offered diets containing nil (■, ▲) or added perennial ryegrass alkaloids (□, Δ) during thermoneutral (squares) or mild heat stress (triangles) conditions. *p*-values for alkaloid, temperature, week effects and interaction between week and temperature, week and alkaloid and week, alkaloid and temperature were <0.001, 0.02, <0.001, 0.41, <0.001, and 0.003 for dry matter intake, 0.64, 0.19, 0.01, 0.63, 0.004, and 0.57 for fecal water, and 0.06, 0.85, 0.10, 0.04, 0.03, and 0.02 for urine output, respectively. The standard error of the difference displayed on the data from Merino sheep receiving nil alkaloid under thermoneutral conditions is for the three-way interaction.

Over the entire three-week period dry matter intake (DMI) was decreased by dietary Alk (701 vs. 442 g/d, *p* < 0.001) and by Heat (712 vs. 443 g/d, *p* < 0.001) (Table 1). Furthermore, there was an Alk × temperature × week interaction (*p* = 0.003, Figure 1a) such that dietary Alk decreased DMI to a greater extent over the latter stages of the study when exposed to Heat. Average daily gain (ADG) was decreased by dietary Alk (−29 vs. −166 g/d, *p* < 0.001) but was not significantly affected by Heat (−77 vs. −118 g/d, respectively, *p* = 0.19). Nevertheless, the lowest ADG (greatest weight loss) occurred in those sheep receiving the combination of dietary Alk and Heat (Table 1). Dry matter digestibility (DMD) was reduced by alkaloid ingestion (67.1% vs. 60.0%, *p* = 0.009). However, there was an alkaloid × temperature interaction (*p* = 0.030) such that the largest reduction in DMD occurred when Alk and Heat were combined (Table 1).

Fecal water increased in the Alk treatment during week 1, and while there was no overall effect of alkaloid on fecal water content (67.5% vs. 68.8%, *p* = 0.64, Figure 1b), fecal water was elevated by dietary Alk during week 1 and thereafter decreased over time (*p* = 0.004). There were no significant effects of dietary Alk or Heat on water intake (Table 1). However, when expressed relative to DMI, water intake increased due to dietary Alk (3.2 vs. 6.2 L/kg DMI, *p* = 0.002) and there was a tendency for urine output to be increased by dietary Alk (0.70 vs. 2.0 L/kg DMI, *p* = 0.06, Figure 1c). There were temperature × week (*p* = 0.04) and Alk × week (*p* = 0.030) and Alk × temperature × week (*p* = 0.020) interactions such that the urine output was relatively low and constant in those sheep consuming the control diet but higher and much more variable in sheep consuming Alk (Figure 1c).

### 2.2. Physiological Responses

**Figure 2:**
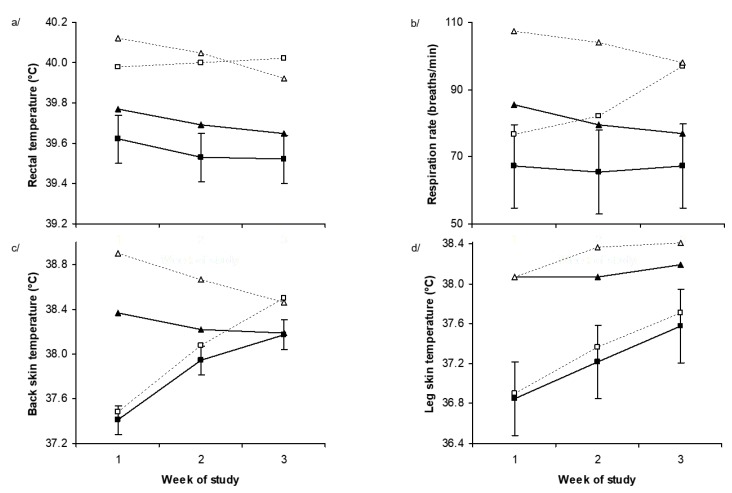
(**a**) Rectal temperature, (**b**) respiration rate, (**c**) back skin temperature, and (**d**) leg skin temperature for each week of study in Merino sheep offered diets containing nil (■, ▲) or added perennial ryegrass alkaloids (□, Δ) during thermoneutral (squares) or mild heat stress (triangles) conditions. *p*-values for alkaloid, temperature, week effects, and the interaction between week and temperature, week and alkaloid, and week, alkaloid and temperature were <0.001, 0.34, <0.001, 0.004, 0.41 and 0.02 for rectal temperature, 0.01, 0.08, 0.57, <0.001, 0.13 and 0.04 for respiration rate, <0.001, <0.001, <0.001, <0.001, 0.98 and 0.006 for back skin temperature, and 0.51, 0.003, <0.001, <0.001, 0.14 and 0.67 for leg skin temperature, respectively. The standard error of the difference displayed on the data from Merino sheep receiving nil alkaloid under thermoneutral conditions is for the three-way interaction.

Overall rectal temperature was increased by dietary Alk (39.63 vs. 40.01 °C, *p* < 0.001) but was not altered by Heat (39.78 vs. 39.87 °C, *p* = 0.34) (Figure 2a). However, there was a temperature × week (*p* = 0.004) interaction such that rectal temperature increased over time in the Heat treatment compared with the TN treatment. Moreover, there was an Alk × temperature × week interaction (*p* = 0.02) which was mainly due to a large increase in rectal temperature in week 1 in the sheep receiving dietary Alk and exposed to high temperatures, followed by a small decrease over time. On the other hand, in those sheep that consumed Alk but were housed at TN temperatures the rectal temperature increased and remained high throughout. Overall, respiration rate was increased by dietary Alk (73 vs. 94 breaths/min, *p* = 0.01) and there was a tendency for respiration rate to increase in the Heat treatment (75 vs. 91 breaths/min, *p* = 0.08, Figure 2b). There was a temperature × week (*p* < 0.001) interaction. Respiration rate increased over time for the Heat treatment compared with the TN treatment, although the responses were variable.

**Figure 3:**
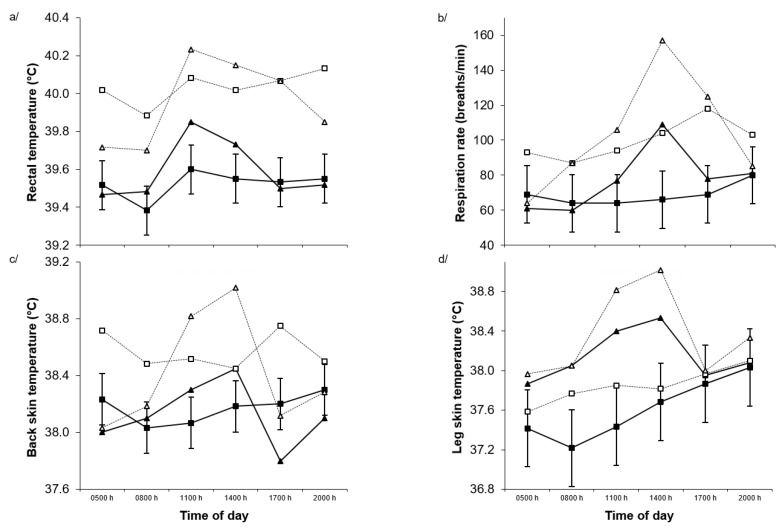
(**a**) Rectal temperature, (**b**) respiration rate, (**c**) back skin temperature, and (**d**) leg skin temperature during the day in Merino sheep offered diets containing nil (■, ▲) or added perennial ryegrass alkaloids (□, Δ) during thermoneutral (squares) or mild heat stress (triangles) conditions over 15 h on day 21 of treatment. *p*-values for alkaloid, temperature and time effects and the interaction between alkaloid and temperature, alkaloid and time, temperature and time, and alkaloid, temperature and time were <0.001, 0.94, <0.001, 0.34, 0.13, <0.001 and 0.17 for rectal temperature, 0.009, 0.51, <0.001, 0.81, <0.001, <0.001 and 0.45 for respiration rate, <0.001, 0.25, <0.001, 0.51, 0.26, <0.001 and 0.09 for back skin temperature, and 0.37, 0.04, <0.001, 0.96, 0.37, <0.001 and 0.18 for leg skin temperature, respectively. The standard error of the difference displayed on the data from Merino sheep receiving nil alkaloid under thermoneutral conditions is for the three-way interaction.

Overall, leg skin temperature was not affected by Alk ingestion (37.66 vs. 37.81 °C, *p* = 0.51, Figure 2d), but was higher in the Heat treatment (37.27 vs. 38.19 °C, *p* = 0.003, Figure 2d). There was a temperature × week (*p* < 0.001) interaction such that temperature increased over time in the Heat treatment compared with the TN treatment. Overall, back skin temperature was higher in the Alk treatment (38.05 vs. 38.35 °C, *p* < 0.001, Figure 3c) and increased in the Heat treatment (37.93 vs. 38.47 °C, *p* < 0.001, Figure 2c). There was a temperature × week (*p* < 0.001) interaction. Temperature increased over time in the Heat treatment compared with the TN treatment. 

Over the fifteen-hour measurement period on day 21, rectal temperature increased in response to dietary Alk (*p* < 0.001) but not Heat (*p* = 0.94) treatment (Figure 3a). However, there was a strong temperature × time (*p* < 0.001) interaction such that rectal temperature was increased to a greater extent over the hot period of the day in those exposed to heat compared to the TN treatment. Respiration rate was increased by Alk (*p* = 0.009) but not Heat (*p* = 0.51) treatment on day 21 (Figure 3b). There were Alk × time (*p* < 0.001) and temperature × time (*p* < 0.001) interactions such that the Alk and Heat treatments increased respiration rate during the hot period of the day while respiration rate remained unchanged in the NilAlk and TN treatments. Back skin temperature was increased by Alk (*p* < 0.001) but not Heat (*p* = 0.25) treatment (Figure 3c). There was a temperature × time (*p* < 0.001) interaction such that Heat treatment increased back skin temperature over the hot part of the day compared to the TN conditions. Leg skin temperature was increased by the Heat treatment (*p* = 0.04) but not by the alkaloid (*p* = 0.37) treatment day 21 (Figure 3d). There was a temperature × time (*p* < 0.001) interaction such that leg skin temperature, over the hot period of the day, was increased to a greater extent in those exposed to heat at this time compared to the TN treatment. 

### 2.3. Plasma Hormones and Metabolites

**Figure 4:**
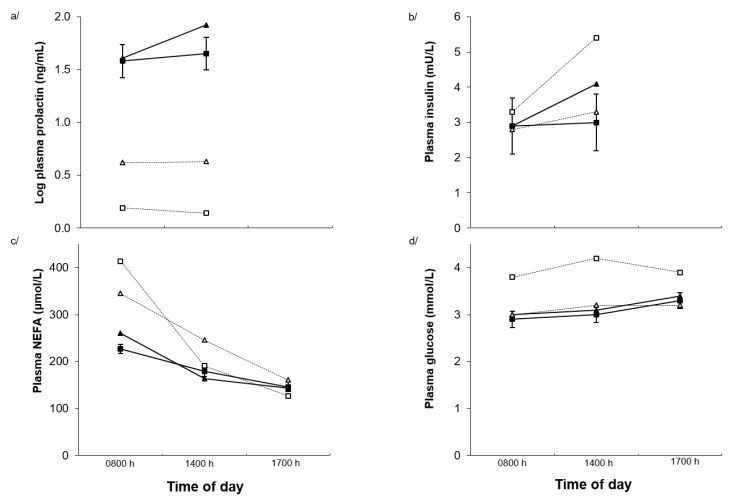
Plasma (**a**) prolactin, (**b**) insulin, (**c**) non-esterified fatty acids (NEFA), and (**d**) glucose concentrations during the day in Merino sheep offered diets containing nil (■, ▲) or added perennial ryegrass alkaloids (□, Δ) during thermoneutral (squares) or mild heat stress (triangles) conditions on day 21 of treatment. *p*-values for alkaloid, temperature and time effects and the interaction between time and temperature, time and alkaloid, alkaloid and temperature, and time, alkaloid and temperature were <0.001, 0.009, 0.080, 0.15, 0.16, 0.041 and 0.35 for plasma prolactin, 0.24, 0.52, 0.020, 0.75, 0.029, 0.42 and 0.10 for plasma insulin, 0.29, 0.74, 0.001, 0.99, 0.64, 0.50 and 0.12 for plasma NEFA, and 0.010, 0.011, 0.50, 0.86, <0.001, 0.25 and 0.26 for plasma glucose concentrations, respectively. The standard error of the difference displayed on the data from Merino sheep receiving nil alkaloid under thermoneutral conditions is for the three-way interaction.

Plasma prolactin was reduced by Alk (48.2 vs. 1.51 ng/mL, *p* < 0.001) and increased by Heat (6.82 vs. 14.8 ng/mL, respectively, *p* = 0.009, Figure 4a). Plasma prolactin concentrations tended (*p* = 0.08) to be higher in the afternoon than in the morning, although there was an Alk × time interaction (*p* = 0.042) such that prolactin concentrations increased in the afternoon only in the sheep on the NilAlk diet. While there were no main effects of Alk (*p* = 0.24) or Heat (*p* = 0.52) alone on plasma insulin concentrations. There was an Alk × temperature interaction (*p* = 0.03) such that Alk treatment increased plasma insulin concentrations under TN conditions but not under heat conditions (Figure 4b). Plasma insulin concentrations were higher (*p* = 0.020) in the afternoon after feeding than in the morning regardless of treatments (Figure 4b). Plasma NEFA concentrations were not altered by Alk ingestion (*p* = 0.29) or Heat (*p* = 0.74) but decreased (*p* < 0.001) in the afternoon (Figure 4c). Plasma glucose concentrations increased in the Alk treatment (*p* = 0.01), but there was no main effect of Heat (*p* = 0.50, Figure 4d). However, there was an Alk × temperature interaction (*p* < 0.001) such that plasma glucose concentrations increased in the Alk treatment under TN conditions. Plasma IGF-1, somatotropin, and leptin concentrations were unchanged by dietary Alk or Heat (Table 2).

## 3. Discussion

One of the most important findings from the present study was that there were clear physiological and production effects of PRG Alk at doses lower than those that induce clinical signs of PRG toxicity (i.e., visible tremors) and that some of these effects will be exacerbated during even mild HS. For example, DMI decreased by 13% in the Heat treatment and by 37% in the Alk treatment and DMI only recovered marginally by the end of the third week. Heat stress in sheep is known to reduce DMI [9]. The magnitude of the decreases, however, was greatest for sheep consuming Alk under mild HS conditions indicating that even moderately elevated temperatures exacerbate the effects of PRG alkaloid ingestion on DMI. This is consistent with a study in cattle in which feed intake decreased the greatest when endophyte-infected tall fescue was fed under 32 °C ambient temperature conditions [10]. Average daily gain was reduced in the Alk treatment but not the heat treatment, presumably due to the greater decrease in DMI in the Alk treatment. In this study, the quality and level of the diet offered were aimed at reflecting conditions when PRG toxicity is likely to occur in the field. Normally, in grazing sheep, liveweight is maintained or reduced under these conditions and this was reflected in the very small to negligible liveweight loss in the NilAlk TN treatment. Liveweight was decreased in sheep grazing infected PRG pasture over summer and autumn [11,12,13], and under controlled Alk intake and thermoneutral conditions [5], although the reasons for this decrease have not been determined. In the current study, sheep reduced DMI after alkaloid ingestion, and therefore, the reduction in ADG is likely a result of this. 

Water intake increased by around 120% in the Alk treatment compared to the control sheep, probably as a mechanism for water conservation to maintain fluid balance and lower heat load [14]. Alternatively, hypertension may be caused by increased heat load, and Alk intake due to the vasoconstrictive action of the ergot alkaloids [15,16], and this could also contribute to increased water intake. Urine output increased in the Alk treatment and this response was consistent with the changes in water intake. However, there was a high degree of variability probably due to differences in the responses of individual sheep to Alk ingestion. A possible explanation of the effects of PRG Alk on urine output may be related to the reduced plasma prolactin concentrations in these sheep. Prolactin may act indirectly on the kidneys, interacting with aldosterone or with antidiuretic hormone causing a decrease in urine output [17,18]. Thus, the decrease in prolactin secretion in the current study may be in part responsible for the increase in urine output observed.

Fecal water increased in the first week of Alk ingestion, although the effect diminished with time possibly due to variation in DMI and/or an adaptation to the PRG Alk. The implications of increased fecal water in sheep in the field are numerous, including increased susceptibility to fly strike, and sub-optimal gastrointestinal function, compromising production and animal welfare [4]. Digestibility decreased in the Alk treatment and this was exacerbated by heat. In a previous study investigating the effects of PRG Alk and severe HS, we observed no effects of Alk on DMD [6], although the dose of both lolitrem B (67%) and ergovaline (27%) was lower than in the present study. Some tall fescue toxicoses studies have found ergovaline decreases DMD [19,20]. There are several factors potentially responsible for a change in DMD which include a direct Alk effect on gastrointestinal function and motility or changes in ruminal flow kinetics [21]. A combination of increased fecal water and decreased DMD could increase the severity of diarrhea and dags and increase the risk of flystrike [4,22]. 

There were moderate increases in respiration rate and rectal temperature in sheep exposed to heat and this is probably due to the relatively low heat load imposed compared to other studies in our laboratory [6,23]. An increase in rectal temperature and respiration rate indicate sheep were under heat load due to PRG Alk consumption which is consistent with a previous study [5] and is thought to be due to altered thermoregulatory responses, including an ergovaline-induced increase in peripheral vasoconstriction. The increase in respiration rate was exacerbated when Alk and mild HS were combined. There was an acute increase in rectal temperature and respiration rate in the sheep consuming Alk and exposed to Heat, which reduced over the experimental period. This could be partly attributed to a reduction in DMI, lowering metabolic heat load. In grazing studies, small increases in rectal temperature and respiration rate over summer and autumn have been observed [4] but the amounts of PRG Alk consumed were unknown. Endophyte infected tall fescue increased rectal temperature and respiration rate in cattle when ambient temperature was 32–35 °C [10].

Plasma prolactin decreased markedly due to Alk ingestion and increased due to heat exposure. Previous studies have reported variable results when sheep were fed PRG or tall fescue Alk although there is generally a reduction [4,13,24]. Prolactin has been found to increase during acute [25] and chronic [26] HS. Prolactin is also thought to play an important role in thermoregulation in sheep [27,28], therefore the suppression of prolactin secretion may compromise thermoregulatory efficiency, along with the well-described effects on reproduction and lactation and potential effects on fluid balance and animal performance [29].

Plasma IGF-1, somatotropin and leptin were not affected by alkaloid ingestion and/or exposure to heat. Plasma insulin increased in sheep during heat exposure in the sheep consuming the control diet indicating that some aspects of metabolism may be altered by increased heat load in sheep. Indeed, differences in plasma glucose were consistent with the differences in plasma insulin such that the lowest plasma glucose concentrations were in the treatments with the higher plasma insulin concentrations. This could be due to DMI decreasing in the combined Alk and Heat treatment reducing the effects of Alk (indirect effect), or due to differences in individual sheep responsiveness to Alk intake. The literature is unclear regarding the effects of Alk on plasma glucose [30,31]. Plasma NEFA concentration was not affected by Alk or Heat. The literature is equivocal with respect to the effects of dietary Alk on plasma NEFA [32,33], although they would be expected to increase when DMI decreases [34].

## 4. Conclusions

Feeding moderate amounts of PRG Alk to sheep under thermoneutral and mild HS conditions can decrease sheep performance. These findings suggest that sheep health and welfare are likely to be compromised when relatively hot ambient temperature conditions occur during summer at a time when pasture Alk concentrations are highest.

## 5. Materials and Methods

This experiment was approved by the University of Melbourne, Science, Optometry and Vision Sciences, and Land and Environment Animal Ethics Committee (Protocol number 1011621.1). Permission approval on 19 March 2010.

All Merino sheep were PR-naïve before the commencement of the experiment. Alkaloids were offered as whole wild-type PRG seed and infected perennial ryegrass hay. Seed was offered whole since in goats only 1.6% of whole perennial ryegrass seed passes through to the feces intact [35]. Sheep were allocated on the basis of liveweight into groups of four and within each group randomly assigned to one of four treatment groups: Two alkaloid concentrations offered × two temperature regimes. The control diet treatment did not receive any PRG seed as there was no guaranteed uninfected seed available. However, the control group did receive PRG hay that was free of alkaloids. The alkaloid treatment (Alk) received whole infected PRG seed and hay at 110 μg/kg LW ergovaline and 75 μg/kg LW lolitrem B. The alkaloid levels offered were aimed at inducing physiological changes (see [5]) but not sufficient to induce clinical PRG toxicity (>100 μg/kg LW lolitrem B, [2]). A small amount of barley grain was mixed with the PRG seed to increase the palatability of the seed. Four replicates of six sheep were rotated through the temperature-controlled rooms. Two replicates were kept at constant thermoneutral (TN) 21–22 °C conditions and two replicates were kept under a heat (Heat) regimen of 33 °C between 1000 h and 1500 h and TN (21–22 °C) for the rest of the day. The temperature–humidity index (THI) was calculated according to the following formula: THI = db °C −[(0.31 − 0.31 RH)(db °C − 14.4)] where db °C is dry bulb temperature in degrees Celsius and RH is relative humidity percentage/100 [9].

The alkaloid feeding level was based on the ergovaline and lolitrem B levels used in a previous study by the same authors [5]. In the current study, infected PRG hay was included in the diet to increase the level of lolitrem B in order to more closely represent the ratio of ergovaline to lolitrem B experienced in the field [1]. Sheep were fed the roughage and seed/barley diet at 08:00 h and 16:00 h. The seed/barley grain mix was offered first to ensure consumption. All sheep were offered the diet at approximately 1.5× maintenance energy levels to reflect likely field conditions and to allow sheep to express differences in DMI. Dry matter intake and water consumption were measured daily. Liveweight was measured weekly in the morning prior to feeding. Urine and fecal separators were placed under the metabolism crates and on day 7 and from day 14 to 24, total feces and urine were collected daily (over 24 h) and urine volume and fecal weight recorded. To determine DM content, subsamples of feces were obtained and dried at 100 °C until samples reached a stable weight usually by 24 h.

Rectal temperature, respiration rate, and skin temperature were measured every three hours from 08:00 h to 17:00 h on days, 3, 6, 8, 10, 13, 15, 17, and 20. A digital thermometer (Vega Technologies Inc., Dongguan City, China) was used to measure rectal and skin (below the spine, above the hip bone, and on the hind leg) temperatures. Respiration rate was calculated by counting the number of breaths taken (flank movements) in 30 s.

Approximately 10 mL of jugular blood was collected via jugular venipuncture on day 21 at 0800 h, 1400 h, and 1700 h and transferred to lithium heparin vacutainers (BD^®^, Franklin Lakes, NJ, USA), centrifuged to collect plasma, which was stored at −25 °C. Metabolite analyses were undertaken on the plasma samples using commercially available kits as follows: Plasma glucose was analyzed with Thermo Infinity glucose oxidase liquid (Thermo Fisher, Noble Park, VIC, Australia), plasma non-esterified fatty acid (NEFA) was analyzed with NEFA-C (Wako Chemicals USA, Richmond, VA, USA) adapted for use in a 96-well microtiter plate system [36]. Hormones were measured by radioimmunoassay (RIA) as follows: Plasma insulin was assayed, as described by [37], insulin-like growth factor (IGF)-1 was assayed using the chloramine-T RIA method, as described by [38], somatotropin was assayed by double-antibody RIA [39]. Plasma leptin was assayed using double-antibody RIA [40], and plasma prolactin was assayed using RIA [41]. Plasma hormones were measured in two samples from each sheep (08:00 h and 14:00 h) whereas all three samples were analyzed for metabolites.

Statistical analysis was performed using Genstat statistical package, 13th Edition (VSN International, Hemel Hempstead, UK) using general analysis of variance. The model including fixed effects of alkaloid (Nil vs. Alk), temperature (TN vs. Heat) and time (hour, day, or week depending on the parameter), the random effect was Sheep ID (when applicable). On day 21, to analyze changes in metabolites and physiological parameters over time, the model included fixed effects of alkaloid, temperature, and time (hour), the random effect was Sheep ID. Where appropriate, covariates were included in the analysis using either baseline data or day 0 data.

## Figures and Tables

**Table 1:** Effect of dietary alkaloids on growth performance, water intake, and dry matter digestibility during thermoneutral and heat conditions.

Alkaloid (Alk)	NilAlk		Alk			*p*-Value		
Temperature (T)	TN	Heat	TN	Heat	Sed	Alk	T	Alk × T
Average daily gain, g/d	−8.0	−50.0	−146	−187	41.6	<0.001	0.19	0.98
Dry matter intake, g/d	717	687	473	413	40.4	<0.001	0.020	0.60
Water intake, L/d	1.97	2.22	2.34	2.74	0.432	0.15	0.44	0.81
Water intake, L/kg DMI	2.99	3.41	5.68	6.85	1.191	0.002	0.36	0.66
Dry matter digestibility, %	66.5	67.8	65.1	55.0	6.70	0.009	0.55	0.030

**Table 2:** Effect of dietary alkaloids on plasma hormone concentrations on day 21 of treatment.

Alkaloid (Alk)	NilAlk		Alk			*p*-Value		
Temperature (T)	TN	Heat	TN	Heat	Sed	Alk	T	Alk × T
IGF-I, ng/mL	25.0 ^1^	25.8	36.2	19.3	10.61	0.76	0.29	0.25
Somatotropin, ng/mL	1.72	2.48	1.60	1.69	0.851	0.98	0.14	0.90
Leptin, ng/mL	1.20	1.30	1.21	1.09	0.092	0.28	0.89	0.23

^1^ Values are the average of blood samples taken at 0800 and 1400 h.
